# Transverse myelitis secondary to Melioidosis; A case report

**DOI:** 10.1186/1471-2334-12-232

**Published:** 2012-09-28

**Authors:** Shanika Nandasiri, Harith Wimalaratna, Muditha Manjula, Enoka Corea

**Affiliations:** 1 Department of Medicine, Teaching Hospital, Kandy, Sri Lanka; 2 Department of Medical Microbiology, Teaching Hospital, Kandy, Sri Lanka; 3 Department of Microbiology, Faculty of Medicine, University of Colombo, Colombo, Sri Lanka

**Keywords:** Melioidosis, Transverse myelitis, Burkholderia pseudomallei, Flaccid paraplegia, Psoas abscess

## Abstract

**Background:**

Melioidosis has become an emerging infection in Sri Lanka; a country which is considered non endemic for it. Paraplegia due to *Burkholderia pseudomallei* is a very rare entity encountered even in countries where the disease is endemic. There are no reported cases of transverse myelitis due to melioidosis in Sri Lankan population thus we report the first case.

**Case presentation:**

A 21 year old farmer presented with sudden onset bi lateral lower limb weakness, numbness and urine retention. Examination revealed flaccid areflexic lower limbs with a sensory loss of all modalities and a sensory level at T_10_ together with sphincter involvement. MRI of the thoracolumbar spine showed extensive myelitis of the thoracic spine complicating left psoas abscess without definite extension to the spinal cord or cord compression. *Burkholderia pseudomallei* was isolated from the psoas abscess pus cultures and the diagnosis of melioidosis was confirmed with high titers of *Burkholderia pseudomallei* antibodies and positive PCR. He was treated with high doses of IV ceftazidime and oral cotrimoxazole for one month with a plan to continue cotrimoxazole and doxycycline till one year. Patient’s general condition improved but the residual neurological problems persisted.

**Conclusion:**

The exact pathogenesis of spinal cord melioidosis is not quite certain except in the cases where there is direct microbial invasion, which does not appear to be the case in our patient. We postulate our patient’s presentation could be due to ischemia of the spinal cord following septic embolisation or thrombosis of spinal artery due to the abscess nearby. A neurotrophic exotoxin causing myelitis or post infectious immunological demyelination is yet another possibility. This emphasizes the necessity of further studies to elucidate the exact pathogenesis in this type of presentations.

Health care professionals in Sri Lanka, where this is an emerging infection, need to improve their knowledge regarding this disease and should have high degree of suspicion to make a correct and a timely diagnosis to reduce the morbidity and mortality due to *Burkholderia pseudomallei* infection. It is highly likely that this infection is under diagnosed in developing countries where diagnostic facilities are minimal. Therefore strategies to improve the awareness and upgrade the diagnostic facilities need to be implemented in near future.

## Background

Melioidosis is an infection caused by *Burkholderia pseudomallei,* a gram negative soil and fresh water saprophyte. It is endemic in tropical and sub tropical zones of South East Asia and northern Australia [1,2]. Even though Sri Lanka has been considered non endemic for melioidosis, there is increasing evidence for its emergence in the recent past. It is likely that melioidosis is under diagnosed in Sri Lanka due to the lack of awareness among health care professionals about the disease because its unfamiliarity and unavailability of the facilities needed for the confirmation [1]. This disease being commoner in rural populations where it goes undetected and the high mortality before a diagnosis is made, may also have contributed to the so far perceived low prevalence of Melioidosis in our country [1].

*Burkholderia pseudomallei* infection is commoner in males and predominantly involves people between 40 to 60 years and is less common in children [2,3]. It spreads by direct inoculation through skin or by inhalation or ingestion. For this reason, it was commonly seen among the soldiers during world wars, as well as in farmers. Diabetes mellitus, chronic lung and renal disease and alcoholism are the common predisposing factors [2]. Melioidosis results in a spectrum of clinical manifestations ranging from asymptomatic disease, localized skin ulcers or abscesses to fulminant disease with disseminated infection with multiple organ involvement. Latent infections caused by this organism are due to its ability to survive intracellularly in phagocytic and non-phagocytic cells for many years while avoiding host immune responses [4]. Along with this, the ability to escape from endocytic vesicles into the cytoplasm and subsequent intracellular replication and cell to cell spread by actin based motility and induction of cell fusion are important characteristics of this infection. Long term survival is maintained by using several defined mutants [4].

It involves almost any organ of the body while lungs being the commonest. Skin, subcutaneous tissue involvement, infections in the urogenital tract, musculoskeletal system, liver and splenic abscess formation were frequently reported [3].

Neurological melioidosis is less common and is seen in 3% of Australian series and results in brain abscesses, meningoencephalitis, brain stem encephalitis and rarely transverse myelitis [2,3,5-8]. A 20 year prospective study carried out in northern Australia where the disease is said to be endemic, had come across 14 patients with central nervous system melioidosis and out of them there had been only two patients with myelitis [8]. There are no published data in literature of melioidosis presenting as transverse myelitis in Sri Lankans. Thus we report the first case of acute flaccid paralysis due to *Burkholderia pseudomallei* infection in Sri Lankan population.

## Case presentation

A 21 year old farmer from North Central province of Sri Lanka; presented to Teaching Hospital- Kandy, with sudden onset bi lateral lower limb weakness and numbness with urine retention. Three days prior to this event he had had a low grade fever with chills, which subsided without medication. There was no history suggestive of skin ulceration or of trauma. His past medical history had been uneventful and he was a teetotaler and a non smoker. As a paddy farmer he had had repeated exposure to water and soil at work.

On examination, he was afebrile and the skin examination was normal. His pulse rate was 80 bpm with a blood pressure of 120/80 mmHg. Respiratory system and abdomen were clinically normal. Neurological examination revealed flaccid areflexic lower limbs with power of grade zero. He had sensory impairment of all modalities including joint position sensation and vibration; with a sensory level at T _10_ and involvement of sphincters. His upper limbs, cranial nerves and higher functions were normal.

**Figure 1:**
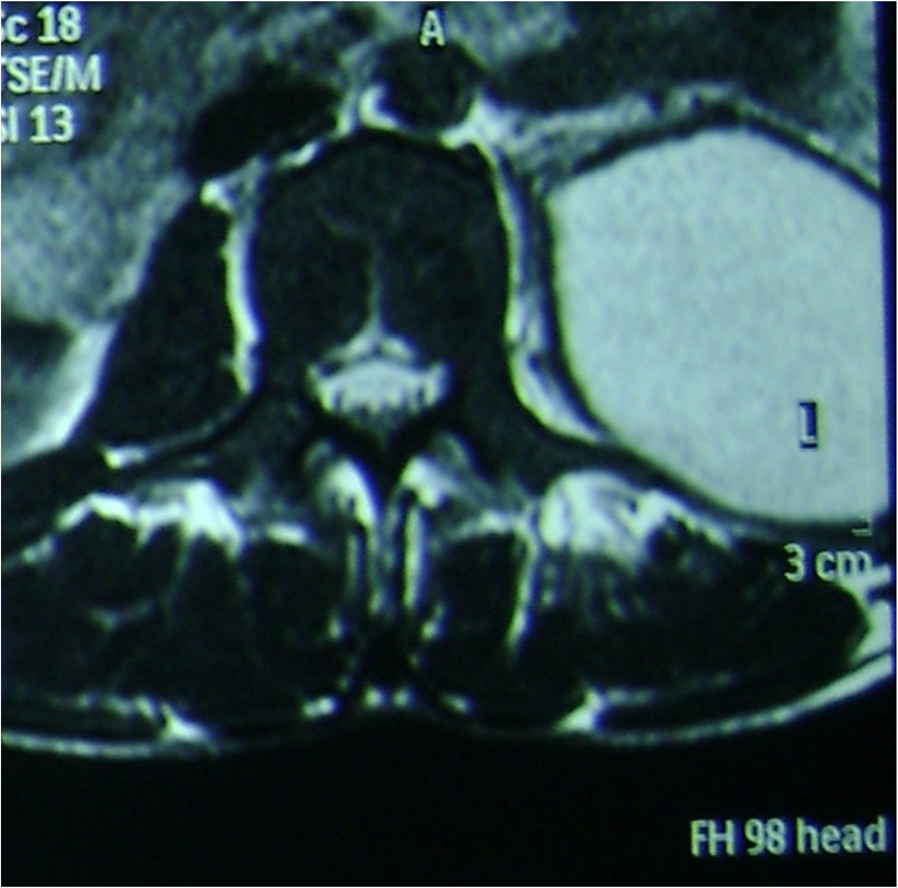
CT scan dorsal spine showing left psoas abscess.

**Figure 2:**
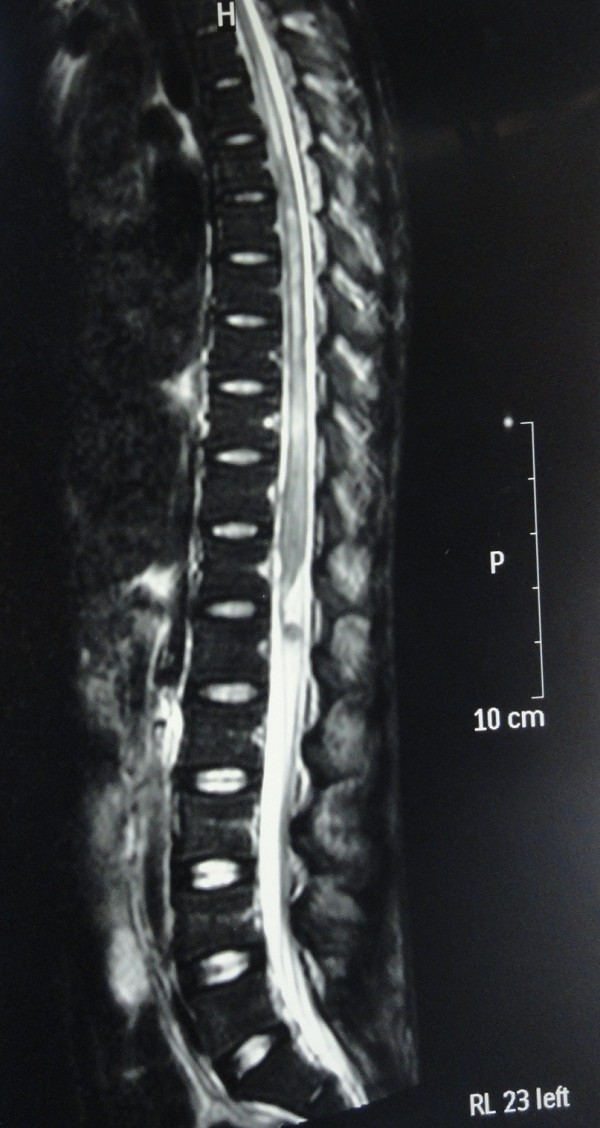
**MRI thoracolumbar spine showing extensive myelitis.** MRI thoracolumbar spine shows extensive involvement of the spinal cord from T4 downwards with foci of T2W hyperintensity and meningeal enhancement.

His full blood count showed a neutrophil leukocytosis. (White blood cell count - 20.91 × 10^9^, 89% Neutrophils), Erythrocyte sedimentation rate was 120/1^st^ hour and C- reactive protein was 196 mg/dl. His X-ray and Computerized tomography (CT) of the dorsal spine was normal, however CT dorsal spine incidentally revealed an abscess in the left psoas muscle (Figure 1). Magnetic resonance imaging (MRI) scan abdomen and thoracolumbar spine revealed a large multi loculated psoas abscess measuring 29.7 cm × 5.7 cm, which involves the entire length of the left psoas muscle with finger like extensions towards the spine. There was extensive myelitis involving the spinal cord from T4 downwards with foci of T2 weighted (T2W) hyperintensity and meningeal enhancement but definite extension of abscess to the spinal canal or a cord compression could not be seen. There was central canal dilatation in the entire length of the spinal cord (Figure 2). There were no other foci of sepsis noted.

An urgent ultrasound guided aspiration of the psoas abscess was carried out and about 350 cc of pus drained. A sample of pus was grown in both blood agar and Macconkey agar (MA). On blood agar it formed medium sized whitish non haemolytic colonies and on Macconkey agar it was typical non lactose fermenting colonies (NLF). Most of the culture characteristics which are needed for the presumptive identification of *Burkholderia pseudomallei* were present. When MA plate was kept in room temperature for 24 hours, it gave lactose fermenting colony appearance. The characteristic earthy smell too was present. (Though smelling of the plate is not recommended). When the isolate was gram stained, gram negative bacilli with characteristic safety pin appearance were seen. Biochemical identification was done using API (Analytical Profile Index)-20NE kit. Its diagnosis was given as *Burkholderia pseudomallei*. Antibiotic susceptibility testing was done by CLSI (Clinical and Laborotary Standards Institute) method. They were sensitive to Ceftazidime and resistant to Gentamicin and Colistin. Diagnosis of melioidosis was made due to the presence of high titres of *Burkholderia pseudomallei* antibodies and positive polymerase chain reaction (PCR). PCR was done with Burk Lpxo PCR Primer sequence. [BURK-3 F (Forward) 5’- GCG CCG CTC AAT TG TTT C -3’ and BURK-2R (Reverse) 5’- CCA CTC GCG CTT GAG GA].

His blood and urine cultures were negative and the fasting and post prandial sugar levels were normal. His renal function tests, liver function tests, coagulation screen and chest X ray were normal. CT Scan of the head was normal but CT scan of the chest was not performed. Repeat ultrasound scan of the abdomen confirmed complete evacuation of the psoas abscess. Cerebrospinal fluid (CSF) studies in this patient was not attempted because of the risk of introducing organisms into the central nervous system as the MRI could not rule out a definite extension of abscess to the spinal cord. Viral studies to exclude other causes of transverse myelitis were not performed because of the financial constrains and the patient being a previously healthy male, the rarity of existence of dual infections.

He was treated with IV ceftazidime 2 g 8 hourly (120 mg/kg/day) and oral cotrimoxazole 1920 mg twice daily for a period of one month followed by a course of oral cotrimoxazole 1920 mg twice daily together with oral doxycycline 100 mg twice daily until one year. As a treatment for myelitis, three day course of IV methylprednisolone 1 g daily was also continued after aspirating the abscess. Physiotherapy, bowel and bladder care and routine care to prevent pressure sores were arranged from the first day of the illness. Following completion of the course of IV antibiotics patient was transferred to a rehabilitation hospital for supportive care. Even though there was no progression of the illness, the residual neurological deficits including the paraplegia, complete sensory loss and sphincter disturbance persisted.

## Conclusion

Our patient had a psoas abscess and transverse myelitis. Even though the presentation was an acute paralysis, he may have acquired the infection sometime prior to this presentation with gradual asymptomatic progression to a brief septicaemic phase with subsequent localization to psoas muscle. The exact pathogenesis of spinal cord melioidosis is not quite certain except in the cases where there is direct microbial invasion, which does not appear to be the case in our patient [6,7]. Probable hypotheses for this presentation would be direct compression of the spinal cord from the psoas abscess or vascular compromise of spinal cord secondary to thrombosis or septic embolisation from the infected psoas muscle or due to exotoxin induced myelitis and demyelination. However, there was no direct invasion or compression of the spinal cord from the psoas absecess which was evident radiologically. Complete cord involvement without arterial territory specific changes makes vascular compromise less likely, however, an angiographic study would have been more helpful. Therefore, we presume exotoxin induced myelitis or demyelination would be the most likely cause of paralysis in our case. This emphasizes the necessity of further studies to elucidate the exact pathogenesis in these type of presentations. Melioidosis can involve any organ system of the body and may present with varying unusual clinical manifestations. High degree of suspicion is, therefore needed to make a proper and a timely diagnosis to prevent the disastrous consequences of the illness especially in a country like Sri Lanka, which is situated in the endemic belt of melioidosis.

Low endemicity of Melioidosis in our country may well be due to the under diagnosis because of its unfamiliarity to the clinical and lab staff of the health care centers. Being a developing third world country, scarcity of the essential diagnostic facilities also contributes to the above problem. Strategies to improve the awareness and diagnostic facilities need to be implemented in the near future in order to minimize the morbidity and mortality associated with this condition in our country.

## Consent

Written informed consent was obtained from the patient for publication of this Case report and any accompanying images. A copy of the written consent is available for review by the Series Editor of this journal.

## Abbreviations

CT: Computerized tomography; MRI: Magnetic resonance imaging; T2W: T2 weighted; PCR: Polymerase chain reaction; T _10_: 10^th^ Thoracic dermatome; MA: Macconkey Agar; NLF: Non Lactose Fermenting.

## Competing interests

The authors declare that they have no competing interests.

## Authors' contributions

HW made the clinical diagnosis and supervised the manuscript drafting. SN drafted the manuscript, reviewed the literature and involved in direct management of the patient. MM carried out the microbiological diagnosis. EC carried out the PCR testing. All authors read and approved the final manuscript.

## Authors' information

HW- MBBS, MD, FRCP(Edin), FRCP(Lond), FCCP - Consultant Physician, Teaching Hospital, Kandy, Sri Lanka.

SN – MBBS – House officer- General Medicine Unit, Teaching Hospital, Kandy, Sri Lanka.

MM – MBBS, MD Medical Microbiology – Senior Registrar in Medical Microbiology, Teaching Hospital, Kandy, Sri Lanka.

EC – MBBS, Diploma in Medical Microbiology, MD Medical Microbiology – Senior Lecturer in Department of Microbiology, Faculty of Medicine, University of Colombo, Sri Lanka.

## Pre-publication history

The pre-publication history for this paper can be accessed here:

http://www.biomedcentral.com/1471-2334/12/232/prepub
